# Editorial: Comprehensive risk prediction in cardiomyopathies: new genetic and imaging markers of risk, volume II

**DOI:** 10.3389/fcvm.2023.1282587

**Published:** 2023-09-13

**Authors:** Nuno Cardim, Luis Rocha Lopes, Giovanni Quarta

**Affiliations:** ^1^ Cardiology Department, Hospital CUF Descobertas, Lisbon, Portugal; ^2^ Nova Medical School, Lisbon, Portugal; ^3^ Barts Heart Centre, St. Bartholomew’s Hospital, London, United Kingdom; ^4^ Institute of Cardiovascular Science, University College London, London, United Kingdom; ^5^ Cardiovascular department, Papa Giovanni XXIII Hospital, Bergamo, Italy

**Editorial on the Research Topic** Comprehensive risk prediction in cardiomyopathies: new genetic and imaging markers of risk, volume II

Risk prediction is relevant to many questions in medicine and specifically in cardiology, and the predicted risk is used to support preventive clinical decisions with the goal of saving lives. However, the success of these initiatives obviously depends on the adequate performance of the risk prediction markers and models (1–3).

The different types of cardiomyopathies collectively represent a substantial burden of disease around the world, causing sudden cardiac death, heart failure and thromboembolism (1). Many of these complications and deaths could be prevented if an adequate risk prediction becomes standard practice.

In the era of precision medicine, risk prediction plays a major role. In patiens with similar clinical presentation, identification of specific characteristics that allow precise definition of prognosis and the selection of preventive individualized strategies is critical. This accurate phenotyping implies the use of “diagnosis tools of precision” such as data from genetics (4) and imaging (5, 6), among others. Data provided by these fine-tuning strategies, when combined with modern data analysis techniques (big data analytics), promise an individualized preventive approach, tailored to the specific characteristics of each patient, with impact on their outcomes.

Cardiomyopathies are important areas of cardiology, in which genetics and cardiac imaging can successfully contribute as “diagnosis tools of precision” to predict risk. Despite recent approaches and advances in this area, risk prediction in these diseases is still far from perfect and clearly there is a lot of room for improvement.

In this research topic of Frontiers in Cardiovascular Medicine, the input of cardiomyopathy investigators on new genetics and imaging markers in comprehensive risk prediction in cardiomyopathies is provided. After the success of the first volume, this new volume introduces interesting new insights on the field.

In a review paper, Galli et al. provide a nice overview of the evolution of cardiac imaging for the assessment of left ventricular dyssynchrony and its role in the selection of patients undergoing cardiac resynchronization therapy. They highlight the main pitfalls and advantages of the application of cardiac imaging for dyssynchrony assessment and provide perspectives for clinical application and future research in this field.

In another elegant paper on dilated cardiomyopathy, Amin et al. demonstrate how genetics and cardiac magnetic resonance frequently change the classification of etiology in dilated cardiomyopathy, improve accuracy and interobserver variability in determining the diagnosis and have an impact on proposed management.

Also on the topic of dilated cardiomyopathy, Zhu et al. show that the “summed motion score” assessed by gated SPECT myocardial perfusion imaging is an independent predictor of cardiac death in these patients and provides incremental prognostic value, challenging the predictive value of left ventricular ejection fraction for early cardiac death.

In Anderson-Fabry disease patients, Parisi et al. discuss the important role of the electrocardiogram in this challenging disease, not only as a sensitive tool for diagnosis, but also for the long-term follow-up of these patients.

Finally, a paper from Zaidi et al. assesses the current role of machine learning in the analysis of complex late gadolinium enhancement patterns to improve risk prediction of major arrhythmic events in stable coronary artery disease patients; these findings highlight a potential novel approach to identifying candidates for implantable cardioverter defibrillators.

## References

[1] Arbelo E Protonotarios A Gimeno JR Arbustini E Barriales-Villa R Basso C 2023 ESC guidelines for the management of cardiomyopathies. Eur Heart J. (2023):ehad194. 10.1093/eurheartj/ehad194 37622657

[2] Writing Committee M, Ommen SR Mital S Burke MA Day SM Deswal A 2020 AHA/ACC guideline for the diagnosis and treatment of patients with hypertrophic cardiomyopathy: a report of the American college of cardiology/American heart association joint committee on clinical practice guidelines. Circulation. (2020) 142:e533–57. 10.1161/CIR.0000000000000938 33215938

[3] Aktaa S Tzeis S Gale CP Ackerman MJ Arbelo E Behr ER European society of cardiology quality indicators for the management of patients with ventricular arrhythmias and the prevention of sudden cardiac death. Europace. (2023) 25(1):199–210. 10.1093/europace/euac114 36753478 PMC10103575

[4] Joy G Moon JC Lopes LR. Detection of subclinical hypertrophic cardiomyopathy. Nat Rev Cardiol. (2023) 20(6):369–70. 10.1038/s41569-023-00853-7 36869094

[5] Quarta G Aquaro GD Pedrotti P Pontone G Dellegrottaglie S Iacovoni A Cardiovascular magnetic resonance imaging in hypertrophic cardiomyopathy: the importance of clinical context. Eur Heart J Cardiovasc Imaging. (2018) 19(6):601–10. 10.1093/ehjci/jex323 29309572

[6] Cardim N Galderisi M Edvardsen T Plein S Popescu BA D’Andrea A Role of multimodality cardiac imaging in the management of patients with hypertrophic cardiomyopathy: an expert consensos of the European association of cardiovascular imaging endorsed by the Saudi heart association. Eur Heart J Cardiovasc Imaging. (2015) 16:280. 10.1093/ehjci/jeu291 25650407

